# Intestinal apicomplexan parasitoses among a hospital-based population in Honduras, 2013-2019

**DOI:** 10.7705/biomedica.6104

**Published:** 2021-12-15

**Authors:** Jorge García, Jackeline Alger, Ramón Jeremías Soto

**Affiliations:** 1 Servicio de Parasitología, Departamento de Laboratorio Clínico, Hospital Escuela, Tegucigalpa, Honduras Departamento de Laboratorio Clínico Hospital Escuela Tegucigalpa Honduras; 2 Maestría en Epidemiología, Departamento de Salud Pública, Facultad de Ciencias Médicas, Universidad Nacional Autónoma de Honduras, Tegucigalpa, Honduras Facultad de Ciencias Médicas Universidad Nacional Autónoma de Honduras Tegucigalpa Honduras; 3 Instituto de Enfermedades Infecciosas y Parasitología Antonio Vidal, Tegucigalpa, Honduras Instituto de Enfermedades Infecciosas y Parasitología Antonio Vidal Tegucigalpa Honduras; 4 Unidad de Investigación Científca, Facultad de Ciencias Médicas, Universidad Nacional Autónoma de Honduras, Tegucigalpa, Honduras Facultad de Ciencias Médicas Universidad Nacional Autónoma de Honduras Tegucigalpa Honduras

**Keywords:** Parasitic diseases, intestinal diseases, cryptosporidiosis, cyclosporiasis, HIV infections, case-control studies, Honduras, enfermedades parasitarias, parasitosis intestinales, criptosporidiosis, ciclosporiasis, infecciones por HIV, estudios de casos y controles, Honduras

## Abstract

**Introduction:**

Intestinal apicomplexa protozoa are a recognized cause of gastroenteritis. They are endemic in Honduras and their epidemiology varies in different population groups.

**Objective:**

To identify risk factors for cyclosporiasis, cryptosporidiosis, and cystoisosporiasis.

**Materials and methods:**

We conducted a case-control study in a hospital-based population. We performed the diagnosis using the modifed Ziehl-Neelsen staining technique and collected the information from laboratory records and clinical charts.

**Results:**

Cyclosporiasis was associated with diarrhea (OR=2.28; 95%CI: 1.10-4.89), weight loss (OR=12.7; 95%CI: 2.49-122.00), watery stools (OR=2.42; 95%CI: 1.26-4.65), and infection with another protozoan (OR=3.13; 95%CI: 1.66-5.95). Cryptosporidiosis was associated with HIV infection (OR=15.43; 95%CI: 3.34-71.22), diarrhea (OR=3.52; 95%CI: 1.40-9.40), lymphopenia (OR=6.16; 95%CI: 1.99-18.98), and green color stools (OR=3.00; 95%CI: 1.23-7.30). Cystoisosporiasis was associated with HIV infection (OR=11.20; 95%CI: 3.53-35.44), diarrhea (OR=7.30; 95%CI: 1.89-28.52), leukopenia (OR=4.28; 95%CI: 1.33-13.75), green color stools (OR=11.59; 95%CI: 1.16-558.60), and Charcot-Leyden crystals (OR=11.59; 95%CI: 1.16-558.60).

**Conclusions:**

In this hospital-based population from Honduras, HIV infection was a risk factor for cryptosporidiosis and cystoisosporiasis, but not for cyclosporiasis.

## Resumen

**Introducción.:**

Los protozoos Apicomplexa intestinales son causa reconocida de gastroenteritis. Estas parasitosis son endémicas en Honduras y su epidemiologia varia según los grupos poblacionales.

**Objetivo.:**

Identifcar los factores de riesgo para ciclosporiasis, criptosporidiosis y cistoisosporiasis.

**Materiales y métodos.:**

Se hizo un estudio de casos y controles en población hospitalaria. El diagnóstico se hizo utilizando la coloración modifcada de Ziehl-Neelsen. La información se obtuvo del registro de laboratorio y las historias clínicas.

**Resultados.:**

La ciclosporiasis se asoció con diarrea (OR=2,28; IC_95%_ 1,10-4.89), pérdida de peso (OR=12,7; IC_95%_ 2,49-122), heces líquidas (OR=2,42; IC_95%_ 1,26-4,65), infección con otros protozoos (OR=3,13; IC_95%_ 1,66-5,95). La criptosporidiosis se asoció con el HIV (OR=15,43; IC_95%_ 3,34-71,22), la diarrea (OR=3,52; IC_95%_ 1,40-9,40), la linfopenia (OR=6,16; IC _95%_ 1,99-18,98), las heces de color verde (OR=3,00; IC_95%_ 1,23-7,30). La cistoisosporiasis se asoció con el HIV (OR=11,20; IC_95%_ 3,53-35,44), la diarrea (OR=7,30; IC_95%_ 1,89-28,52), la leucopenia (OR=4,28; IC_95%_ 1,33-13,75), las heces de color verde (OR=11,59; IC_95%_ 1,16- 558,60), y los cristales de Charcot-Leyden (OR=11,59; IC_95%_ 1,16-558,60).

**Conclusiones.:**

En este estudio de base hospitalaria en Honduras, el HIV fue un factor de riesgo para la criptosporidiosis y la cistoisosporiasis, pero no así para la ciclosporiasis.

Intestinal apicomplexan protozoa (*Cyclospora cayetanensis, Cryptosporidium spp.,* and *Cystoisospora belli*) are an important cause of gastroenteritis in the world, especially in low-income countries (LIC). *Cyclospora cayetanensis* infects subjects of all ages and its presence is associated with the rainy season and outbreaks where the transmission occurs through raw food and contaminated water ^1^. On the other hand, *Cryptosporidium* spp. is common among children less than five years old and is one of the main causes of diarrhea ^2^^,^^3^; in the adult population, the infection is associated with immunosuppression ^4^. Risk factors such as poverty, lack of sanitation, contact with animals, and poor-quality water have been described ^5^. In most cases, *C. belli* is detected among immunosuppressed populations with HIV infection and is considered an opportunistic pathogen, although cases have also been described among immunocompetent subjects, as well as some risk factors such as lack of sanitation and poor-quality water ^6^.

These parasitoses are endemic in Honduras where the first reports go back to 1986 ^7^. Since then, they have been described several times among patients hospitalized in *Hospital Escuela* in Tegucigalpa ^8^^-^^11^, which has taken to the systematic search of the parasites in stool samples, but analytical studies are needed to better understand their local epidemiology.

We conducted the present study to identify risk factors associated with intestinal apicomplexan parasitoses and describe their distribution by age and sex, their clinical presentation, and the laboratory data among the patients from a public hospital in Honduras.

## Materials and methods

We conducted a case-control study among patients attending the parasitology service at *Hospital Escuela* clinical laboratory in Tegucigalpa, Honduras, for six years from January, 2013, to December, 2019. Intestinal apicomplexan protozoa oocysts were identified in stool samples using the modified Ziehl-Neelsen staining technique; then, they were measured with a microscope with a micrometer eyepiece ^12^. Additionally, we analyzed all stool samples by direct wet smear with saline and iodine solution. The Parasitology Service has laboratory technicians, a laboratory technologist, and a parasitologist (Ph.D.) as permanent staff to carry out and supervise the parasitological diagnoses, and textbooks, bench aids, and positive samples/ slides are available for reference. The search was done in all ambulatory or hospitalized patients five or under five years of age, as well as in five-year- olds and older individuals with watery or loose stools and/or by medical request. Study subjects were also retrospectively identified from laboratory records. One stool sample was analyzed for each subject.

Cases were defined as those patients with oocysts and single apicomplexa infection whose clinical records were available at *Hospital Escuela*. Controls were subjects with no intestinal apicomplexa oocysts. The controls were matched by age (± 2 years) and sex with the cases in a 2:1 ratio. We established three groups according to the parasite identified: *Cyclospora, Cryptosporidium*, or *Cystoisospora*.

To determine the strength of the association with possible exposure factors, we calculated odds ratios (OR) and their 95% confidence intervals (95% CI). To evaluate the statistical significance, we used chi-square and Fisher tests while the Mann-Whitney U test was used for median comparison. Microsoft Excel and the OpenEpi online statistical calculator were used to perform these analyzes (http://www.openepi.com/Menu/OE_Menu.htm).

The study protocol was approved by the ethics committee from the medical sciences school at *Universidad Nacional Autónoma de Honduras* (UNAH), (IRB 00003070 Office of Human Research Protection, https://ohrp.cit.nih gov/search/IrbDtl.aspx.). We also obtained authorization from the Registration Department and the Directorate of Teaching and Research at *Hospital Escuela* to access the clinical records.

Our study was based on the review of laboratory records and clinical charts. There was no direct interaction with the patients and, therefore, it was not necessary to obtain informed consent or assent. Contact information (names, telephone, ID number, etc.) were not recorded to minimize a potential loss of confidentiality and each data collection instrument was assigned an identification code.

## Results

From 2013 to 2019, we searched for the presence of apicomplexan protozoa in 10,938 patients; we identified 131 (1,2 %) cases of *Cyclospora cayetanensis* infection, 94 (0.9%) of *Cryptosporidium* spp., and 50 (0.5%) of *C. belli*. Using the data from the clinical charts available, we distributed 61 cases and 121 controls in the *Cyclospora* group; 53 cases and 86 controls in the *Cryptosporidium* group, and 27 cases and 52 controls in the Cystoisospora group. Table 1 shows the age and sex proportion between cases and controls in each group; 51.6% of cases in the *Cyclospora* group were male with a median age of 9.5 years; 54.7% of cases in the *Cryptosporidium* group were males with a median age of 4 years, and 51.9% cases in Cystoisospora group were male and the median age was 32 years. We found no statistically significant differences by sex or age among cases and controls within each group, which was expected given the study design.

**Table 1:** Proportional distribution of cases and controls by sex and age according to parasitosis. *Hospital Escuela*, Tegucigalpa, 2013-2019

Variable		*Cyclospora*		*Cryptosporidium*		*Cystoisospora*	
		Cases (n=64) %	Controls (n=121) %	Cases (n=53) %	Controls (n=86) %	Cases (n=27) %	Controls (n=52) %
Sex							
	Male	51.6	48.8	54.7	65.1	51.9	57.7
	Female	48.4	51.2	45.3	34.9	48.1	42.3
Chi square		p=0.8352		p=0.2988		>p=0.7973	
Age (years)							
	< 1	1.5	5.8	18.9	26.7	3.7	5.8
	1 - 5	43.8	41.3	37.7	43.0	3.7	1.9
	6 - 15	9.4	14.0	5.7	3.6	0.0	1.9
	> 15	45.3	38.8	37.7	26.7	92.6	90.4
	Median	9.5	8.0	4	2.5	32.0	36
	(IR)	(3.0 - 23.5)	(2.0 - 24.0)	(1.0 - 34.0)	(0.8 - 20.7)	(27.0 - 45.0)	(30 - 47.2)
Mann- Whitney U		p= 0.6213		p=0.7840		p=0.6423	

IR: Interquartile range

Table 2 describes the variables in the *Cyclospora* group. The main differences observed between cases and controls were living in a house with concrete floors (55.2% vs. 30.8%), adobe walls (44.8% vs. 10.3%), block or brick walls (51.7% vs.71.8%), metal roof (62.1% vs.72.2%), piped water service (77.8% vs. 83.8%), sewerage service (53.6% vs. 71.8%), and having a dog in the house (54.2% vs. 38.9%). The main differences in the clinical presentation were diarrhea (75.5% vs. 57.4%), abdominal pain (42.9% vs. 34.7%), weight loss (20.4% vs. 2.0%), HIV infection (14.0% vs. 4.6%), leukocytosis (18.9% vs. 30.5%), neutrophilia (16.7% vs. 32.5%), thrombocytosis (30.6% vs. 13.4%), watery stools (42.2% vs. 23.1%), fat visible under the microscope (14.1% vs. 4.9%), and infection with other protozoa (53.1% vs. 26.4%).

**Table 2:** Characteristics of cyclosporiasis cases and their controls, *Hospital Escuela*, Tegucigalpa, 2013-2019

Variable			Cases N=64 n (%)	Controls N=121 n (%)	OR	CI95%	p
House building materials							
	Floor		n=29	n=39			
		Concrete	16 (55.2)	12 (30.8)	2.77	1.02 - 7.52	0.0762
		Brick	9 (31.0)	23 (59.0)	0.31	0.11 - 0.86	0.0416
		Soil	4 (13.8)	4 (10.3)	1.40	0.23 - 8.24	0.9355*
	Walls		n=29	n=39			
		Block/Brick	15 (51.7)	28 (71.8)	0.42	0.15 - 1.15	0.1490
		Adobe	13 (44.8)	4 (10.3)	7.10	2.0 - 25.24	0.0029
		Roof	n=29	n=36			
		Metal	18 (62.1)	26 (72.2)	0.63	0.22 - 1.79	0.5463
		Tile	9 (31.0)	7 (19.4)	1.80	0.59 - 5.83	0.4303
	Water source		n=27	n=37			
		Pipe service	21 (77.8)	31 (83.8)	0.67	0.19 - 2.38	0.7766
		Others (well, river, etc.)	6 (22.2)	6 (16.2)	1.47	0.42 - 5.20	0.7766
	Sewerage		n=28	n=39			
		Sewerage	15 (53.6)	28 (71.8)	0.45	0.16 - 1.25	0.1547
		Latrine	12 (42.9)	10 (25.6)	2.10	0.77 - 6.13	0.2023
		None	1 (3.6)	1 (2.6)	1.40	0.02 - 113.3	>0.999*
	Origin		n=61	n=116			
		Tegucigalpa city	39 (63.9)	74 (63.8)	1.00	0.53 - 1.92	0.8839
		Francisco Morazán	7 (11.5)	15 (12.9)	0.87	0.33 - 2.27	0.9687
		Other departments	15 (24.6)	27 (23.3)	1.07	0.52 - 2.21	0.9925
	Animal contact		n=24	n=36			
		Dog	13 (54.2)	14 (38.9)	1.85	0.65 - 5.28	0.3679
		Chicken	5 (20.8)	6 (16.7)	1.30	0.27 - 5.98	0.9341*
		Cat	5 (20.8)	6 (16.7)	1.30	0.27 - 5.98	0.9341*
	Clinical presentation		n=49	n=101			
		Diarrhea	37 (75.5)	58 (57.4)	2.28	1.10 - 4.89	0.0482
		Fever	19 (38.8)	55 (54.5)	0.52	0.26 - 1.06	0.1037
		Vomit	17 (34.7)	53 (52.5)	0.48	0.23 - 0.97	0.0611
		Abdominal pain	21 (42.9)	35 (34.7)	1.41	0.70 - 2.84	0.4271
		Weight loss	10 (20.4)	2 (2.0)	12.7	2.49 - 122.00	0.0005*
	Immunosuppression		n=57	n=108			
		HIV	8 (14.0)	5 (4.6)	3.36	0.90 - 13.60	0.0729*
		Cancer	7 (12.3)	5 (4.6)	2.80	0.74 - 12.05	0.1433*
		Hematological malignancy	8 (14.0)	9 (8.3)	1.79	0.65 - 4.94	0.3808
	Blood cell count		n=38	n=83			
		Leukocytosis	7 (18.9)	25 (30.5)	0.52	0.20 - 1.34	0.2584
		Leukopenia	4 (10.8)	10 (12.2)	0.87	0.18 - 3.31	>0.9999*
		Neutrophilia	6 (16.7)	27 (32.5)	0.39	0.15 - 1.04	0.0893
		Neutropenia	5 (13.9)	7 (8.4)	1.75	0.40 - 6.94	0.5497*
		Lymphocytosis	4 (11.1)	6 (7.4)	1.56	0.30 - 7.08	0.7362*
		Lymphopenia	5 (13.9)	17 (21.0)	0.58	0.19 - 1.73	0.4743
		Anemia	21 (55.3)	44 (53.0)	1.09	0.50 - 2.36	0.9728
		Thrombocytosis	11 (30.6)	11 (13.4)	2.84	1.10 - 7.36	0.0518
	Stool characteristics		n=64	n=121			
		Formed consistency	9 (14.1)	25 (20.7)	0.62	0.27 - 1.44	0.3667
		Soft consistency	12 (18.8)	31 (25.6)	0.67	0.32 - 1.41	0.3847
		Loose consistency	16 (25.0)	37 (30.6)	0.75	0.38 - 1.50	0.5304
		Watery consistency	27 (42.2)	28 (23.1)	2.42	1.26 - 4.65	0.0115
		Mucus	14 (21.9)	39 (32.2)	0.58	0.29 - 1.19	0.1901
		Leukocytes	10 (15.6)	25 (20.7)	0.71	0.32 - 1.59	0.5257
		Fat under the microscope	9 (14.1)	6 (4.9)	3.13	1.06 - 9.25	0.0608
		Charcot-Leyden crystals	2 (3.1)	4 (3.3)	0.94	0.08 - 6.79	>0.9999*
		Other protozoa	34 (53.1)	32 (26.4)	3.15	1.66 - 5.95	0.0006

*Fisher test

*Blastocystis* spp. was the most frequently identified parasite (37.7% vs 19.8%). The participants with a significant probability of being diagnosed or suffering from cyclosporiasis were those living in houses with concrete floor (OR=2.77; 95%IC: 1.02-7.52) and adobe walls (OR=7.1; 95%CI: 2.025.24), as well as those with diarrhea (OR=2.28; 95%CI: 1.10-4.89), weight loss (OR=12.7; 95%CI: 2.49-122), thrombocytosis (OR=2.84; 95%CI: 1.107.36), watery stools (OR=2.42; 95%CI: 1.26-4.65), and coinfection with other protozoa (OR=3.13; 95%CI: 1.66-5.95). On the contrary, subjects who lived in houses with brick walls (OR=0.31; 95%CI: 0.11-0.86) and had vomiting (OR = 0.48; 95%CI: 0.23-0.97) showed a significantly lower probability of being diagnosed with cyclosporiasis.

In the *Cryptosporidium* group (table 3), the main differences between cases and controls were living in a house with concrete floors (43.5% vs. 21.9%), block or brick walls (52.2% vs. 58.1%), metal roof (59.1% vs. 65.6%), piped water service (69.9% vs. 90.0%), sewerage service (60.0% vs. 57.1%), and having a dog in the house (39.1% vs. 29.2%). As for the clinical presentation, the main differences between cases and control were diarrhea (80.4% vs. 53.8%), fever (65.2% vs. 51.3%), cough (21.7% vs. 15.4%), HIV infection (30.0% vs. 2.7%), leukopenia (37.5% vs. 9.7%), lymphopenia (35.9% vs. 8.3%), anemia (65.0% vs. 53.2%), green color stools (28.3% vs. 11.6%), loose stools (24.5% vs. 33.7%), watery stools (28.3% vs. 20.9%), stools with mucus (52.8% vs. 30.2%), and the presence of Charcot-Leyden crystals (3.8% vs. 1.2%). Subjects with a significantly higher probability of being diagnosed or suffering from cryptosporidiosis were those with HIV infection (OR=15.43; 95%CI: 3.34-71.22), a history of diarrhea (OR=3.52; 95%CI: 1.40-9.40), leukopenia (OR=5.60; 95%CI: 1.90-16.12), lymphopenia (OR=6.16; 95%CI: 1.99-18.98), green color stools (OR=3.00; 95%CI: 1.23-7.30), and stools with mucus (OR=2.58; 95%CI: 1.27-5.25).

**Table 3:** Characteristics of cryptosporidiosis cases and their controls. *Hospital Escuela*, Tegucigalpa, 2013-2019

Variable			Cases N=53 n (%)	Controls N=86 n (%)	OR	CI95%	p
House materials building							
	Floor		n=23	n=32			
		Concrete	10 (43.5)	7 (21.9)	2.74	0.85 - 8.9	0.1574
		Brick	5 (21.7)	16 (50.0)	0.28	0.08 - 0.93	0.0648
		Soil	7 (30.4)	9 (28.1)	1.10	0.34 - 3.62	0.9085
	Walls		n=23	n=31			
		Block/Brick	12 (52.2)	18 (58.1)	0.78	0.26 - 2.33	0.8777
		Adobe	8 (34.8)	10 (32.2)	1.12	0.35 - 3.50	0.9225
	Roof		n=22	n=32			
		Metal	13 (59.1)	21 (65.6)	0.75	0.24 - 2.31	0.8401
		Tile	4 (18.2)	6 (18.7)	0.96	0.17 - 4.76	>0.9999*
	Water source		n=23	n=30			
		Pipe service	16 (69.6)	27 (90.0)	0.25	0.04 - 1.34	0.1266*
		Others (well, river, etc.)	7 (30.4)	3 (10.0)	3.94	0.89 - 17.42	0.1266*
	Sewerage		n=20	n=28			
		Sewerage	12 (60.0)	16 (57.1)	1.12	0.35 - 3.61	0.9212
		Latrine	6 (30.0)	12 (42.9)	0.57	0.17 - 1.92	0.3453
	Origin		n=49	n=85			
		Tegucigalpa city	26 (53.1)	58 (68.2)	0.52	0.25 - 1.08	0.1179
		Francisco Morazán	8 (16.3)	10 (11.8)	1.46	0.53 - 3.99	0.6292
		Other departments	15 (30.6)	17 (20.0)	1.76	0.78 - 3.95	0.2397
	Animal contact		n=23	n=24			
		Dog	9 (39.1)	7 (29.2)	1.50	0.46 - 5.26	0.6798
		Chicken	8 (34.8)	5 (20.8)	2.00	0.54 - 7.48	0.4578
		Cat	1 (4.3)	4 (16.7)	0.22	0.004 - 2.62	0.3741*
	Clinical presentation		n=46	n=78			
		Diarrhea	37 (80.4)	42 (53.8)	3.52	1.40 - 9.40	0.0054
		Fever	30 (65.2)	40 (51.3)	1.78	0.84 - 3.78	0.1856
		Vomit	20 (43.5)	36 (46.2)	0.89	0.43 - 1.86	0.9184
		Abdominal pain	10 (21.7)	16 (20.5)	1.07	0.44 - 2.62	0.9471
		Cough	10 (21.7)	12 (15.4)	1.50	0.60 - 3.80	0.5148
	Immunosuppression		n=50	n=74			
		HIV	15 (30.0)	2 (2.7)	15.43	3.34 - 71.22	0.00005
		Cancer	5 (10.0)	3 (4.1)	2.63	0.48 - 17.62	0.3419*
		Hematological malignancy	5 (10.0)	5 (6.8)	1.53	0.33 - 7.05	0.7420*
	Blood cell count		n=40	n=62			
		Leucopenia	15 (37.5)	6 (9.7)	5.60	1.9 - 16.12	0.0016
		Leukocytosis	10 (25.0)	19 (30.6)	0.75	0.31 - 1.84	0.6948
		Neutrophilia	5 (13.2)	16 (26.7)	0.42	0.14 - 1.25	0.1703
		Neutropenia	5 (13.2)	7 (11.7)	1.14	0.26 - 4.59	>0.9999*
		Lymphocytosis	12 (30.8)	8 (13.3)	2.89	1.05 - 7.90	0.0617
		Lymphopenia	14 (35.9)	5 (8.3)	6.16	1.99 - 18.98	0.0016
		Anemia	26 (65.0)	33 (53.2)	1.60	0.71 - 3.70	0.3319
		Thrombocytosis	8 (20.0)	14 (22.9)	0.83	0.32 - 2.23	0.9499
	Stool characteristics		n=53	n=86			
		Green color	15 (28.3)	10 (11.6)	3.00	1.23 - 7.30	0.0239
		Formed consistency	4 (7.5)	11 (12.8)	0.55	1.16 - 1.84	0.4925
		Soft consistency	21 (39.6)	28 (32.6)	1.35	0.66 - 2.76	0.5067
		Loose consistency	13 (24.5)	29 (33.7)	0.63	0.29 - 1.37	0.3390
		Watery consistency	15 (28.3)	18 (20.9)	1.49	0.67 - 3.29	0.5853
		Mucus	28 (52.8)	26 (30.2)	2.58	1.27 - 5.25	0.0132
		Leukocytes	11 (20.7)	20 (23.2)	0.86	0.37 - 1.98	0.8932
		Fat under the microscope	4 (7.5)	13 (15.1)	0.45	0.14 - 1.48	0.2923
		Charcot-Leyden crystals	2 (3.8)	1 (1.2)	3.33	0.16 - 198.90	0.6481*
		Other protozoa	7 (13.2)	16 (18.6)	0.53	0.18 - 1.58	0.5507

*Fisher test

In the *Cystoisospora* group (table 4), we were not able to collect data about house characteristics. The main differences between cases and controls were diarrhea (87.5% vs. 48.8%), vomit (58.3 vs. 39.0%), abdominal pain (41.7% vs. 19.5%), HIV infection (77.8% vs. 23.8%), leukopenia (43.5% vs. 16.3%), lymphopenia (52.2% vs. 15.7%), and anemia (78.3% vs. 51.2%). Cases presented with stools of green color (18.5% vs. 1.9%), watery stools (44.4% vs. 30.8%), mucus (48.1% vs. 44.2%), fat visible under the microscope (18.5% vs. 1.9%), and Charcot-Leyden crystals (18.5% vs. 1.9%) more frequently than controls. Subjects with the most statistically significant probability of being diagnosed or suffering from cystoisosporiasis were those with HIV infection (OR=11.20; 95%CI: 3.53-35.44), diarrhea (OR=7.30; 95%CI: 1.89-28.52), leukopenia (OR=4.28; 95%CI: 1.33-13.75), anemia (OR=3.43; 95%CI: 1.0810.93), green color stools (OR=11.59; 95%CI: 1.16-558.60), microscopically visible fat (OR=11.59; 95%CI: 1.16-558.60), and the presence of Charcot- Leyden crystals (OR=11.59; 95%CI: 1.16-558.60). Subjects with leukocytosis had a significantly lower probability of suffering cystoisosporiasis (OR=0.12; 95%CI: 0.003-0.92).

**Table 4:** Characteristics of cystoisosporosis cases and their controls. *Hospital Escuela*, Tegucigalpa, 2013-2019

Variable		Cases N=27 n (%)	Controls N=52 n (%)	OR	CI95%	^p^
Origin		n=26	n=49			
	Tegucigalpa city	17 (65.4)	23 (46.9)	2.13	0.79 - 5.71	0.2006
	Francisco Morazán	2 (7.7)	7 (14.3)	0.50	0.05 - 2.94	0.6640*
	Other departments	7 (26.9)	19 (38.8)	0.58	0.20 - 1.64	0.4404
Animal contact		n=6	n=7			
	Dog	3 (50.0)	3 (42.9)	1.33	0.09 - 9.13	>0.999*
	Chicken	1 (16.7)	3 (42.9)	0.27	0.004 - 5.49	0.6853*
	Cat	1 (16.7)	1 (14.3)	1.20	0.012 - 9.70	>0.999*
Clinical presentation		n=24	n=41			
	Diarrhea	21 (87.5)	20 (48.8)	7.30	1.89 - 28.52	0.004
	Fever	11 (45.8)	17 (41.5)	1.19	0.43 - 3.29	0.9332
	Vomit	14 (58.3)	16 (39.0)	2.20	0.78 - 6.10	0.2120
	Abdominal pain	10 (41.7)	8 (19.5)	2.90	0.96 - 9.03	0.1012
Immunosuppression		n=27	n=42			
	HIV	21 (77.8)	10 (23.8)	11.20	3.53 - 35.44	0.00003
	Cancer	1 (3.7)	2 (4.7)	0.76	0.02 - 15.64	>0.9999*
	Hematological malignancy	1 (3.7)	4 (9.5)	0.37	0.007 - 4.02	0.6892*
Blood cell count		n=23	n=43			
	Leukocytosis	1 (4.3)	12 (27.9)	0.12	0.003 - 0.92	0.0380*
	Leukopenia	10 (43.5)	7 (16.3)	4.28	1.33 - 13.75	0.0346
	Neutrophilia	2 (9.1)	13 (32.5)	0.20	0.04 - 1.02	0.0927
	Neutropenia	2 (9.1)	3 (7.5)	1.23	0.09 - 11.68	0.9999*
	Lymphocytosis	1 (4.3)	2 (2.4)	0.86	0.01 - 17.53	0.99999*
	Lymphopenia	12 (52.2)	13 (15.7)	2.26	0.79 - 6.48	0.1377
	Anemia	18 (78.3)	22 (51.2)	3.43	1.08 - 10.93	0.0597
	Thrombocytosis	4 (18.2)	5 (12.2)	1.60	0.27 - 8.41	0.7688*
Stool characteristics		n=27	n=52			
	Green color	5 (18.5)	1 (1.9)	11.59	1.16 - 558.60	0.0323*
	Formed consistency	1 (3.7)	9 (17.3)	0.18	0.004 - 1.28	0.1599*
	Soft consistency	8 (29.6)	6 (11.5)	3.22	0.83 - 12.77	0.0960*
	Loose consistency	6 (22.2)	21 (40.4)	0.42	0.14 - 1.22	0.1727
	Watery consistency	12 (44.4)	16 (30.8)	1.80	0.68 - 4.70	0.3384
	Mucus	13 (48.1)	23 (44.2)	1.17	0.46 - 2.97	0.9255
	Leukocytes	4 (14.8)	10 (25.0)	0.52	0.15 - 1.79	0.8596
	Fat under the microscope	5 (18.5)	1 (1.9)	11.59	1.16 - 558.60	0.0320*
	Charcot-Leyden crystals	5 (18.5)	1 (1.9)	11.59	1.16 - 558.60	0.0323*
	Other protozoa	5 (18.5)	15 (28.8)	0.56	0.17 - 1.75	0.4663

* Fisher test

## Discussion

Some of the risk factors for these parasitoses were poverty and lack of drinking water and sewerage services ^5^^,^^6^^,^^13^^-^^15^. These conditions are prevalent in Honduras and they may explain our findings. Living in a house with brick floors might protect against cyclosporiasis (and possibly against cryptosporidiosis), which may be a proxy indicator of better socioeconomic status. On the other hand, living in a house with mud walls was associated with cyclosporiasis, which might indicate lower socioeconomic status but was not measured in this study. We observed differences in the frequency of piped water service, sewerage, and the presence of domestic animals between cases and controls in the *C. cayetanensis* and *Cryptosporidium* spp. groups. However, these findings were not statistically significant.

We found an association between cyclosporiasis, diarrhea, and weight loss. These results are consistent with the clinical presentations described for cyclosporiasis in other studies including diarrhea, abdominal pain, fatigue, weight loss, and vomiting both in children and adults ^16^^,^^17^. Watery stools and the presence of other protozoans were associated with cyclosporiasis in this study. Similar observations have been made in Honduras relating watery stools to the clinical presentation ^11^ andthe presence of other protozoa coinfections in other studies ^16^ probably explained by the similar transmission modes of *C. cayetanensis* and other commensal protozoans.

Diarrhea was associated with *Cryptosporidium* cases, which is consistent with other studies ^17^^-^^19^. The proportion of cough was higher among the cases (21.7%) compared to the controls (15.4%) in the *Cryptosporidium* group while respiratory symptoms were not frequent among cyclosporiasis and cystoisosporiasis cases. Studies in Uganda showed that coughing was present in 78% of immunocompetent children with cryptosporidiosis while oocysts were identified in the sputum of 35.4% of children with cough ^20^. In adults, respiratory cryptosporidiosis was present in 1.3% of subjects with HIV infection and 4.4% of HIV-negative individuals with suspected tuberculosis ^21^. In our study, coughing was not statistically associated with cryptosporidiosis but the results could point to a possible respiratory infection. Unfortunately, the systematic search for this parasite was not conducted despite the presence of coughing.

In our study, diarrhea was associated with *C. belli* cases and we observed that fever, vomiting, and abdominal pain were frequent among cases but with no statistical association, which is consistent with the clinical presentation already described ^6^: diarrhea with abdominal pain, nausea, fever, and weight loss.

We found that subjects with HIV infection were 15 times more likely to be infected by *Cryptosporidium* spp. and 11 times by *C. belli* but HIV infection was not a risk factor for *C. cayetanensis*. These findings are consistent with observations previously made in Honduras where *Cryptosporidium* spp. and *C. belli* were more frequent among HIV-infected populations but was not the case of *C. cayetanensis*^10^. Similar observations have been reported in other countries where *Cryptosporidium* spp. and *C. belli* were associated with immunosuppressed people due to HIV, organ transplants, or leukemia ^22^^-^^25^. Our results confirmed that these parasitoses are opportunistic and endemic in immunosuppressed populations, especially those infected with HIV. Thus, the systematic surveillance of these protozoans is highly recommended. Leukopenia and lymphopenia were associated with cryptosporidiosis. Similarly, we found an association with leukopenia and cystoisosporiasis but not with lymphopenia, although it was more frequent among the cases (43.5%) than the controls (16.3%) probably due to immunosuppression by HIV infection, which was frequent among these cases.

We found no association between cryptosporidiosis and stool consistency. On the other hand, a formed consistency of the stools was present in 47% of cases probably because of asymptomatic oocysts shedding after the episode of diarrhea, which in cryptosporidiosis can occur for up to seven months ^26^. It is possible that among our participants, the episode of diarrhea resolved while the asymptomatic period of oocyst shedding remained. Charcot-Leyden crystals observed microscopically in the stool examination were associated with cystoisosporiasis but no association with eosinophilia was found, which would have been expected because these crystals are related to the activity of eosinophils. Some studies have reported cystoisosporiasis associated with eosinophilia and Charcot-Leyden crystals ^27^.

Limitations in our study refer to the diagnostic method. The modified Ziehl- Neelsen staining technique is a cheap and relatively simple laboratory method, although it requires quality reagents, equipment, and human resources, but it is not very sensitive for detecting the infections given the different patterns of parasite excretion or the low density in the stool sample. It has been estimated that this staining technique can detect 78% of cases compared to other more expensive and specialized methods (fluorescence microscopy, immunoenzymatic assays, or molecular biology) ^14^^,^^27^^-^^31^. This could have affected the number of cases detected during this period or, more importantly, the selection of controls. Therefore, the inclusion of false negative controls should not be discarded and, thus, selection bias. Ideally, the use of molecular tools with greater sensitivity is required to detect these infections. Secondly, our sample size represented 51.3% of the total cases in the 2013-2019 period. Besides, the information on the variables was not complete, which reflected in the wide 95% confidence intervals estimated for the ORs and indicates low precision and statistical power, which should be considered when interpreting the results. Thirdly, we did not search for other non-parasitic pathogens causing diarrhea, so we do not know if the cases or the controls had infections other than the parasitoses under study. Additionally, we could not determine if the clinical presentation and laboratory data were risk factors since they were not defined as exposure variables occurring before the parasitosis.

In conclusion, HIV infection was an important risk factor for acquiring *Cryptosporidium* spp. and *C. belli*, but not *C. cayetanensis* among this hospital-based population in Honduras. Diarrhea is part of the clinical presentation of the three parasitoses, but it was only associated with cyclosporiasis. We also found associations with other variables but the study’s low statistical power prevents further conclusions. However, such variables may be risk factors for acquiring these parasitoses, and, therefore, it would be convenient to study them using laboratory detection tools with a higher sensitivity in cohort studies. Although these parasites are endemic in Honduras, further studies are required to better understand the local epidemiology, clinical presentation, circulating *Cryptosporidium* species, and other aspects yet to be explored.
